# An assist for cognitive diagnostics in soccer (Part II): Development and validation of a task to measure working memory in a soccer-specific setting

**DOI:** 10.3389/fpsyg.2022.1026017

**Published:** 2023-01-23

**Authors:** Simon Knöbel, Franziska Lautenbach

**Affiliations:** ^1^Faculty of Sport Science, Chair of Sport Psychology, Leipzig University, Leipzig, Germany; ^2^Sport Psychology, Institute of Sport Science, Humboldt-Universität zu Berlin, Berlin, Germany

**Keywords:** executive functions, diagnostics, soccer performance, validity, talent development

## Abstract

Cognitive diagnostics is of increasing interest to researchers and practitioners in the context of talent identification and performance enhancement in professional soccer. Research addressing the relevance of cognitive skills for sports performance has been based on the cognitive component approach (i.e., general cognitive processes) and the expert performance approach (i.e., sport-specific cognitive processes). Following the aim to combine the strengths of both approaches, we have previously developed and validated tasks to measure inhibition and cognitive flexibility in a soccer-specific setting, including a soccer-specific motor response. In line with the broad consistency on three core executive functions, this further development of diagnosing executive functions is to be completed with a task for the assessment of working memory. For this purpose, 60 amateur players with a soccer experience of at least one competitive season (*M*_age_ = 25.95, *SD*_age_ = 4.59) first conducted a computer-based version of the *n*-back (3-back) task followed by a 3-back task that required a soccer-specific motor response (i.e., pass) performed in a soccer-specific setting (i.e., SoccerBot100). Results show good reliability for both tasks. With regard to convergent validity, significant correlations between the computerized and soccer-specific task could be determined in target trials for response time (*r* = 0.446) and accuracy (*r* = 0.401). Thus, the soccer-specific *n*-back task can be considered a potentially valid instrument for assessing working memory and potentially allows soccer clubs to diagnose the three core executive functions in a consistent soccer-specific setting.

## Introduction

Given complex and multifaceted performance requirements in soccer, cognition can be a crucial factor to achieve and maintain peak performance. Due to the continuous development of the game with decreasing time and space for each player (Carling, 2010; Wallace and Norton, 2014), latest research addressed psychological skills including cognition to optimize performance (Söhnlein and Borgmann, 2018; Beavan et al., 2020). In this context, the core executive functions (EFs)—inhibition, cognitive flexibility, and working memory—are considered crucial for effective and goal-directed behavior (Diamond, 2013). Technological advances in the field of cognitive diagnostics and training, such as Footbonaut (Saal et al., 2015), Helix (Kittelberger, 2018), or the SoccerBot (Heilmann et al., 2021) provide new possibilities to assess cognition within (more) ecologically valid settings. However, so far, examination of reliability and (ecological) validity of newly developed systems and the cognitive tasks implemented through those are still a limitation restricting cognitive diagnostics (Beavan, 2019; Lautenbach et al., 2022). Most cognitive tasks used for diagnostics in the applied field have neither been developed driven by theory nor have they been validated empirically (Memmert, 2019). Consequently, it remains unclear how cognition can be measured appropriately to enable conclusions for on-field performance (Van Maarseveen et al., 2018). In this context, previously used tasks often lack the coupling of perception and action because they did not require a sport-specific motor response (Farrow and Abernethy, 2003; Murr et al., 2018). In a recent meta-analysis, Kalén et al. (2021) emphasize the application of sport-specific stimuli and responses within cognitive diagnostics to detect expertise related differences.

We have already aimed to implement these requirements in two empirical studies (Musculus et al., 2022). We successfully adapted well-established computerized tasks (see cognitive component approach, Nougier et al., 1991) for inhibition (i.e., flanker task) and cognitive flexibility (i.e., number-letter task), and transferred them to a soccer-specific setting (i.e., SoccerBot360), including soccer-specific stimuli (i.e., soccer players for the soccer-specific inhibition task) and a soccer-specific motor response (i.e., passing the ball; see expert performance approach, Ericsson, 2003). In doing so, we combined the merits of relevant approaches in cognitive diagnostics in sports (for detailed description of both approaches, see Voss et al., 2010; Musculus et al., 2022). In order to complete the previously developed soccer-specific tasks^1^ for inhibition and cognitive flexibility and thus, to cover all core EFs, the aim of this study is to develop and validate a task to measure soccer-specific working memory.

From a theoretical and an applied perspective, investigating working memory (also referred to as updating) is relevant for performance in sports. Especially in strategic sports such as soccer (e.g., Voss et al., 2010), players are confronted with a large amount of information that they have to process and take into account for the selection of their options (Verburgh et al., 2014). Working memory enables individuals to keep information in mind and retrieving this information in order to mentally work with it, even if it “no longer perceptually present” (Diamond, 2013, p. 142). In other words, working memory is the ability to constantly store and update information depending on its relevance to the given situation. Based on this definition, working memory and inhibition are closely linked and rarely occur independently of each other since constant mental retention of the goal is presumed for inhibitory performance (Diamond, 2013). Transferred to competitive soccer, the relevance of working memory results from constant processing and updating of retrieved information. Players must be able to adjust their behavior based on the information gathered and the comparison with previous experiences (Vestberg et al., 2012), for example, if an opponent repeatedly makes the same run or movement. In addition, the players have to keep in mind their tactical setup or instructions, as well as the behavior of their teammates, and adapt them in case of situational changes (Huijgen et al., 2015). Thus, in addition to inhibition and cognitive flexibility, the core EF working memory also have a potential impact on game performance and are thus, also relevant to assess from an applied perspective.

## The present study

In preparation for the study, we identified the *n*-back task (Kirchner, 1958) as a commonly used task to assess working memory capacities (Conway et al., 2005; see Supplementary Table 1A for justification based on previous studies). Additionally, the *n*-back task is highly practicable for the implementation in the SoccerBot due to the merely visual representation of the stimuli and the button press response in the computer task, in comparison to other tasks that require verbal responses (e.g., working memory span test, Conway et al., 2005; Scharfen and Memmert, 2021). At this point, it should be noted that other tasks are also used to assess working memory and correlation with soccer performance has been demonstrated (e.g., Backward Visual Memory Span, Huijgen et al., 2015; Corsi-block task, Scharfen and Memmert, 2019; and design fluency, Vestberg et al., 2017). However, the decision to use the *n*-back task is primarily, regardless of the soccer context, because it is a standard, frequently used and validated task to measure working memory (Kane et al., 2007, p. 615).

The study was conducted in accordance to our preceding soccer-specific task development and evaluation of inhibition and cognitive flexibility. Amateur soccer players performed both computerized and soccer-specific *n*-back tasks. Soccer-specific means that the tasks were conducted in a setting, in which participants were standing and responding to soccer-related stimuli (i.e., pictures of typical soccer actions) by executing soccer-specific responses, namely passing to goals (Musculus et al., 2022). This environment can be considered context-specific for the sample of amateur soccer players and thus, should increase ecological validity of the task (see review by Lumsden et al., 2016). However, while the selected images depict typical soccer actions, they do not represent situations based on which a decision or action must be made during a game. Since the study aims to assess the working memory performance of soccer players in combination with a soccer-specific motor response, the presentation of a representative game situation was less relevant than presenting clearly distinguishable stimuli in the sense of the original task.

We expected to find positive correlations with regard to response times and correct answers in the computer-based task and the soccer-specific task, indicating that the two tasks are related in terms of convergent validity and the soccer-specific tasks also allows for measuring working memory.

Additionally, we collected data on players’ subjective perceptions of fun, stress, motivation, and physical exhaustion due to the tasks to control for potential confounders on cognitive performance. Thus, we asked the players about their physical exhaustion in order to monitor physical load of the participants before and during testing based on studies showing a decline in performance due to cognitive or physical fatigue (e.g., Smith et al., 2016). Along with this, we asked for perceived stress induced by the tasks, also to identify possible differences between the settings. Furthermore, we assessed motivation prior to the respective tasks as it is attributed a potential influence within cognitive diagnostics (Beavan et al., 2020; Vestberg et al., 2020). Finally, the perceived fun of the tasks was assessed based on the assumption that participants’ engagement depends on the motivation and enjoyment elicited by the cognitive task (e.g., Lumsden et al., 2016). Accordingly, the engagement of the participants is an important characteristic of the quality of cognitive data collection (e.g., Walton et al., 2018).

## Materials and methods

### Participants

A total of 60 male soccer players (*M*_age_ = 25.95, *SD*_age_ = 4.59) participated in the study. Only adult players with a soccer experience of at least one competitive season in soccer were included to ensure basic soccer technical skills (see also Musculus et al., 2022). On average, participants had played soccer for 14.82 years (*SD* = 6.03) and practiced 4.07 h/week (*SD* = 7.40). Seven participants used to play in a youth academy when they were younger. Most players played in the seventh (*n* = 17) and sixth highest league in Germany (*n* = 12), followed by nine players in the eighth and seven in the ninth division. Further, three players played in the 10th highest, three in the fifth highest, two in the fourth highest, and one in the third highest league. Prior to participation, all players signed written informed consent. The study was carried out following the Declaration of Helsinki and approved by the ethics committee of Leipzig University (2020.11.17_eb_69).

### Material

#### *n*-Back task

In the *n*-back task, participants see an emotional neutral stimuli (i.e., each presentation of a stimulus is referred to as a trial) and have to decide whether the same stimulus was presented *n* items before (Jaeggi et al., 2010, p. 394). Therefore, a button press response usually on a keyboard is only required if a so-called target trial is presented. If the stimulus *n* positions before does not match the current stimulus and it is therefore a non-target trial the participants should not react, meaning not to press a button or play a pass the ball in the SoccerBot. If a motor response is made to a non-target trial it represents a false alarm.

**Figure 1:**
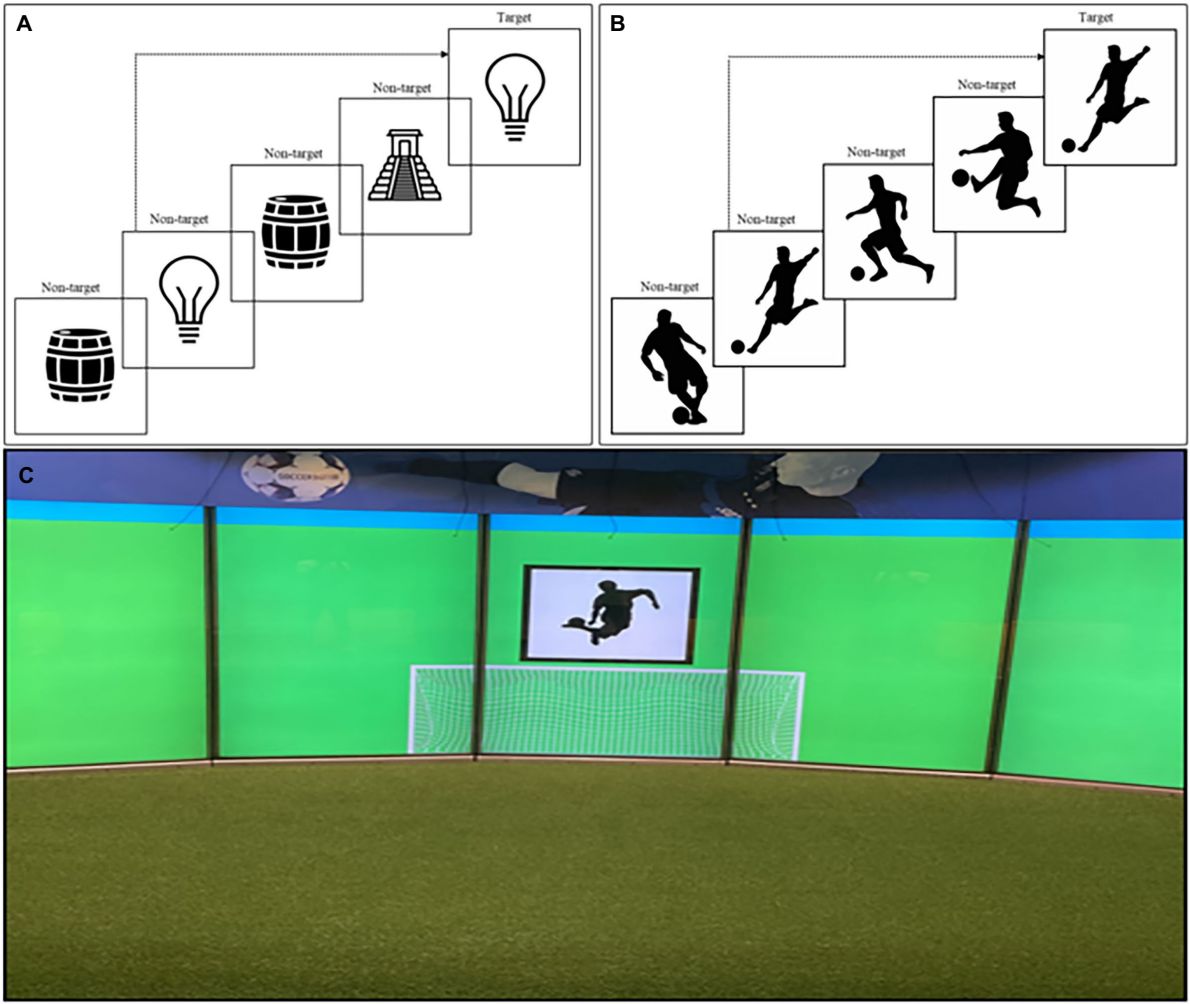
Applied stimuli in the computerized 3-back task **(A)**, adapted for the SoccerBot100 **(B)** and the provided soccer-specific setting **(C)**. Images of SoccerBot reproduced with permission from Umbrella Software.

In the present study, the *n* was set to 3 based on research results that have shown that the 3-back task can be considered the most reliable (Hockey and Geffen, 2004). The main process measured is the updating ability of working memory, which involves the continuous assimilation of new information and the replacement of old information (Hockey and Geffen, 2004; Jaeggi et al., 2010). The collected data show whether and how often the *n*-back was recognized correctly (i.e., accuracy). Updating ability is then evaluated in combination with the required response time. High amount of correct responses and faster response times, represent better working memory (Kirchner, 1958). For the computerized task, stimuli were presented in the form of pictures with various neutral objects, such as a lamp, chair, or bicycle (see Figure 1A).

For the 3-back task developed and used in the SoccerBot100, setting, response, and stimuli were soccer-specific: Players were standing in a soccer field; they had to respond by passing a ball; and pictures of different soccer-related actions were presented as stimuli (see Figure 1B). Participants had to kick the ball into a goal that was constantly presented centered below the changing stimuli (Figure 1C). In case of a target trial, the players should react as quickly as possible with a pass into that goal.

#### Instruments to measure general and sport-specific working memory

To measure general working memory, we presented the computerized cognitive task on a 15-in. Laptop (1,280 × 960 pixels at 60 Hz) at a viewing distance of approximately 60 cm, using Inquisit 5 (2018). Participants were asked to press the key “A” only responding to target trials and not reacting to non-target trials.

Similar to the previous tasks assessing inhibition and cognitive flexibility, the development of the soccer-specific 3-back task was accomplished in collaboration with sport psychologists and sport scientists of a German first division soccer club as well as the Umbrella Software Company. Programmers of the latter institution then implemented this task for the SoccerBot100. The SoccerBot100 is a smaller version of the SoccerBot360 with a smaller field (i.e., artificial grass area) but with walls for projections (for a more detailed description see Supplementary material). In both tasks, participants performed two consecutive experimental blocks. Both blocks of the 3-back task consisted of 50 trials (see justification in Supplementary Table 1A). The ratio of non-target and target trials was set at 70% (35 trials) to 30% (15 trials) per Block oriented on previous studies that have addressed the validity of the computerized *n*-back task (Miller et al., 2009; Jaeggi et al., 2010). Further details of the general and soccer-specific task are presented in Table 1.

**Table 1:** Comparison of general computer *n*-back task and soccer-specific *n*-back task.

Variable	*n*-back task for working memory	
	Computerized	Soccer-specific
No. practice trials	One block with 10 trials	Two blocks with 17 trials
	(Two targets, eight non-target)	(Five targets, 12 non-target)
Feedback practice trials	No	Yes (after played pass)
No. test trials	100 (30 targets, 70 non-targets)	100 (30 targets, 70 non-targets)
No. test blocks (trials per block)	2 (50)	2 (50)
Break between blocks	30 s	30 s
Response correspondence	Fingertip on keyboard (button “A” for targets; no button press for non-targets)	Pass to goal under stimuli for targets: no pass for non-targets
Stimulus presentation	10 different images of various, non-specific objects (e.g., lamp, chair, or bicycle)	10 different images (silhouettes) of soccer-specific actions (e.g., duel, shot, and dribble)
Randomization of presented stimuli	Yes	No
Fixator (fixation duration)	Black screen without Fixator (2,500 ms)	Black + (1,500 ms)
Response–stimulus interval	Trial presented for the shape of 500 ms, another 2,500 ms before presenting the next shape	Trial presented for 2,500 ms, Inter-Stimulus-Interval (1,500 ms)
Response time out	3,000 ms	4,000 ms
Cronbach’s alpha(reaction time)	0.72	0.78
Split-Half Reliability(reaction time)			0.72	0.78

#### Control variables

Motivation of the participants was assessed as a control variable. Accordingly, they answered the question “How motivated are you at this moment?” on a visual analog scale (VAS; Crichton, 2001) with two endpoints from 0 (not at all) to 100 (highly) before performing the computerized and SoccerBot100 tasks. VAS are commonly used to assess moods, stress, or emotions. Within the scope of such investigations, the reliability and validity of the VAS have also been confirmed (e.g., Pfennings et al., 1995). In addition, perceived physical exertion (RPE) of the participants was assessed using the 15-point Borg scale for ratings of perceived exertion (Borg, 1970). The RPE scale is a frequently used measurement that is considered valid based on determined high correlations of the ratings and different physiological variables such as heart rate (Borg, 1982).

Finally, we assessed perceived fun and perceived stress regarding the tasks after players performed them. Players were asked to answer the questions “How much fun did you have doing the current task?” and “How stressful did you find the task?” on a VAS scale from 0 (none) to 100 (a lot) following the respective task. All scales were presented in digital form on a tablet.

### Procedure

For the final experiment, players were recruited through inquiries with regional amateur teams and announcements at the university. The experiment was conducted in the laboratory of the Department of Movement Neuroscience at Leipzig University and lasted approximately 30 min for each player. The study followed a cross-sectional approach with a within-subject design.

First, participants were asked to fill out demographic and soccer-specific questionnaires. This was followed by the general 3-back task on the computer. After that, participants were asked to warm up individually for 5–10 min to reduce the risk of injuries, before starting the sport-specific 3-back task in the SoccerBot100. The assessment of player’s motivation and perceived exertion was conducted before participants performed the tasks to control for potential differences in motivation and physical load during the experiment as well as possible preload due to training or work before testing that could have influenced test performance. At this point, it is important to note that the order of the versions (i.e., first computer task and second soccer-specific task) was fixed in order to familiarize the players with each task and reduce the number of practice trials, and therefore the physical load, in the SoccerBot100 (see Musculus et al., 2022).

### Data preparation

For the computerized and for the soccer-specific 3-back task, a filter was used to identify all missed target trials (PC: 35.79%; soccer-specific: 33.80%). In addition, all responses to non-target trials (i.e., false alarms) were determined (PC: 9.82%, soccer-specific: 5.71%) followed by the exclusion of the corresponding response times. Thus, the mean response time is calculated only based on target trials that the participants correctly identified and responded to. In a second filter, for the computerized task, all trials with response times lower than 200 ms or higher than 3,000 ms (0.0%) were excluded (e.g., Lautenbach et al., 2016). For the soccer-specific task, the same filter (0.0%) was used but with 400 ms as the lower bound due to longer responses times for the whole-body movement (Morya et al., 2003). In addition, a third filter excluded all response times that deviated ±3 *SD* from the individual mean were excluded (computerized 0.0%, soccer-specific 0.0%). Both filters were applied to control for extreme results caused by, for example, speculating or waiting too long, thus being inattentive to the task.

In order to calculate the overall accuracy in a first step, the percentage of correct answers to target trials and the percentage of false alarms were determined. Then, the percentage of correct target trials (e.g., 70%) minus the percentage of incorrect answers for non-targets (i.e., false alarms, 10%) was calculated (e.g., 60% overall accuracy). Additionally, we investigated the percentage of missed target trials (e.g., 30%) as this provides a measurement of how many correct answers went into the analysis of response time, which is only measured based on correct responses to target trials. This additional measure is relevant as some athletes might be fast in their response time but only a small number of trials were answered correctly.

Overall, three players had to be excluded because of incomplete data sets (two in the computerized task because only half of all trials were recorded, one in the soccer-specific task because incomplete data collection). Thus, the analyses included a total of 57 participants.

### Data analyses

The dependent variables were first checked for normality and outliers. For both tasks, accuracy and response time parameters were normally distributed. Two outliers were detected for the 3-back task with regard to accuracy values in the general computerized task. For the soccer-specific task in the SoccerBot100, no outliers were detected. All data were analyzed using SPSS Statistics, version 25. Initially, the level of significance was set at *p* < 0.05 for all analyses.

First, to control for potential influences of motivation and perceived exhaustion, we checked whether there was a difference in motivation or perceived exhaustion prior to the computerized and soccer-specific task by running two paired *t*-tests. If differences were found, we followed up by calculating Pearson correlations with the dependent variables of accuracy and response time.

To test convergent validity for the soccer-specific task (Hypothesis 1), we calculated Pearson correlations between response time and accuracy for the general, computerized task, and the adapted soccer-specific version. In addition, we controlled for potential learning effects by calculating paired *t*-tests for response times and accuracy between block 1 and block 2 within the respective tasks. Finally, we ran dependent *t*-test to assess the fun and stress participants perceived during the two versions of the tasks.

## Results

Statistical analyses indicated the same pattern of results when outliers were included and thus, all analyses are reported including the outliers. The descriptive statistics for response times and accuracy for the computerized general and soccer-specific versions of the 3-back task are shown in Table 2. Reliability, assessed *via* split-half reliability (coefficient *r*) and Cronbach’s Alpha for response time parameters, show high values for both computerized general (*r* = 0.72; *α* = 0.72) and soccer-specific task (*r* = 0.78; *α = 0*.78).

**Table 2:** Descriptive statistics for the general, computerized, and soccer-specific versions of the tasks measuring working memory (*n* = 57).

Task	Descriptive statistics			
	*M*	*SD*	Min	Max
Computerized				
RT, target trials (ms)	970.2	231.61	601.6	1,505
Overall accuracy, all trials (%)	51.44	15.02	10.91	88.48
False alarms (%)	9.82	5.2	2.86	32.86
Missed targets (%)	35.78	13.47	0	70
Soccer-specific				
RT, target trials (ms)	1542.95	192.9	1117.06	1871.73
Overall accuracy, all trials (%)	60.18	15.02	20.95	90
False alarms (%)	5.66	3.74	0	15.71
Missed targets (%)	33.8	13.76	6.67	63.33

RT, response time.

### Convergent validity

For the computerized and the soccer-specific working memory tasks, correlational analyses revealed significant positive correlations for response time (*r* = 0.446, *p* < 0.001) and overall accuracy (*r* = 0.401, *p* < 0.001). The analyses regarding the missed targets confirm the correlations (*r* = 0.352, *p* < 0.001) as well as the false alarms (*r* = 0.409, *p* < 0.001). Further results of correlational analyses are presented in Table 3.

**Table 3:** Correlations for response time and accuracy values between computerized and soccer-specific 3-back task.

Computerized task	Variable	Soccer-specific task				RT_target trials	Acc_all trials	Acc_false alarms	Acc_missed targets
	RT_target trials	0.446^**^	−0.079	0.00	0.102
	Acc_all trials	−0.180	0.401^**^	−0.242	−0.366^**^
	Acc_false alarms	0.072	−0.079	0.409^**^	−0.018
	Acc_missed targets	0.176	−0.357^**^	0.109	0.352^**^

RT, response time; Acc, Accuracy. ^*^p < 0.05; ^**^p < 0.001.

### Control variables (motivation, perceived exhaustion, stress, and perceived fun)

Players reported to be significantly higher motivated prior to the computerized general (*M* = 77.93, *SD* = 16.24) in comparison to the soccer-specific task (*M* = 68.28, *SD* = 21.78), *t*(56) = 4.13, *p* < 0.001, *d* = 0.548. A Pearson correlation revealed significant positive correlations between motivation before the soccer-specific task and response times in the soccer-specific task (*r* = 0.288, *p* = 0.030).

The perceived exhaustion was significantly lower prior to the general computerized task (*M* = 20.51, *SD* = 19.13) in comparison to the soccer-specific task (*M* = 34.77, *SD* = 24.26), *t*(56) = 4.9, *p* < 0.001, *d* = 0.654. A Pearson correlation revealed significant negative correlations between response times and perceived exhaustion (*r* = −0.289, *p* = 0.030) in the SoccerBot100. No significant differences in perceived stress were shown after the computerized (*M* = 60.14; *SD* = 24.20) and the soccer-specific task (*M* = 54.35, *SD* = 25.58), *t*(56) = 1.79, *p* = 0.078, *d* = 0.237.

Finally, participants perceived the task in the SoccerBot100 to be significantly more fun (*M* = 73.56, *SD* = 22.09) than the computerized task (*M = *44.47*, SD = 25*.*22*)*, t*(56) = 8.86, *p* < 0.001, *d* = 0.436. A Pearson correlation, however, did not show any significant correlations between perceived fun and performance in the computerized or soccer-specific task.

### Learning effects

For the computerized task, no significant differences in response time was found between the first (*M* = 992.70, *SD* = 267.29) and the second block (*M* = 950.34, *SD* = 247.59), *t*(56) = 1.32, *p* = 0.190, *d* = 0.185. This also applies to the mean accuracy values in the first (*M* = 49.84%, *SD* = 19.13%) and second block (*M* = 53.03%, *SD* = 19.39%), *t*(56) = 1.01, *p* = 0.314, *d* = 0.134.

Similar results were shown in the soccer-specific task. For response times, no differences were found between the first (*M* = 1527.33, *SD* = 216.08) and second half (*M* = 1555.57, *SD* = 209.65) of the test runs, *t*(56) = 1.18, *p* = 0.240, *d* = 0.156. Also, no differences were found for accuracy (*M_1st block_* = 59.04%, *SD_1st block_* = 17.91%; *M_2nd block_* = 61.32%, *SD_2nd block_* = 15.39%), *t*(56) = 1.01, *p* = 0.371, *d* = 0.134.

## Discussion

Following the previous development of tasks to assess inhibition and cognitive flexibility with soccer-specific stimuli and soccer-specific motor response (i.e., pass), the aim of this study was to develop and validate a task measuring working memory (updating) in the same setting. For convergent validity of the general and the soccer-specific task, we found significant positive correlations for response time as well as accuracy parameters of both tasks. These results as well as the also acceptable values of the reliability indicate that the adapted 3-back task is applicable to measure working memory in adult soccer players.

In general, the use of the *n*-back task to determine individual differences in working memory has been controversially discussed (Jaeggi et al., 2010). Due to weak correlations with performance in other working memory tasks, this discussion mainly focused on the question of whether the *n*-back task only measures working memory (Kane et al., 2007; Miller et al., 2009). Given the dynamic nature of the task (Frost et al., 2021) and complexity of EFs (Miyake et al., 2000), researchers also highlight the potential impact of additional processes. For example, Miller et al. (2009, p. 716) concluded, that “*n*-back accuracy may rely more on information processing speed or motor speed than on working memory…,” independent of the applied *n*-back load that was investigated (1-, 2-, and 3-back loads).

Against the background of a complex performance structure, it can be assumed that working memory performance in soccer is also subject to these processes. Players must not only be able to retrieve and update information, but also adapt their (motor) actions. It is possible that these demands are also represented by the *n*-back task, since studies with elite soccer players show correlations with soccer performance. It was shown that scores from the *n*-back task correlated with goals scored during the season as well as a superiority of elite athletes over athletes and non-athletes (Vestberg et al., 2017; Holfelder et al., 2020).

With respect to the significant but moderate correlations detected for response time and response accuracy for the assessment of convergent validity, the differences between the tasks must be considered. Although the soccer-specific task was based on the computer-based task and is supposed to measure the same construct, there are methodological differences that might limit higher correlations between the tasks. With the implementation in a different environment as well as the differences in terms of response modality and presented stimuli, relevant parameters such as the duration of the stimuli and the time for response were also adjusted. It is possible that these aspects provoke different response behavior. In this context, common method variance (Bryman, 1989) could also be present. This describes variance caused by the measurement method itself rather than the constructs that the measurements represent. Additionally, response format and the general context of the applied methods (Podsakoff, 2003) differ between the computerized and soccer-specific task, despite the fundamentally same construct that the tasks measure (i.e., working memory).

Under closer examination of the results of both tasks, it is noticeable that the accuracy in the soccer-specific task is considerably higher. This may be due to habituation or learning effects between tasks, as the task in the SoccerBot was always performed second. However, at least within the tasks, there were no indications of learning effects. Another explanation would be the longer presentation of the stimuli in the soccer-specific task due to the more complex motor responses. This is consistent with studies showing that image recognition, as well as numerical discrimination accuracy, increases with longer stimulus duration (Bird and Cook, 1979). In this context, it has been shown that information reception as well as memorizing of stimuli is facilitated by longer presentation through repeated recall (Inglis and Gilmore, 2013; Pergher et al., 2020). With respect to the current soccer-specific task, it might also be plausible to assume that better accuracy might be due to the more relatable stimuli (i.e., soccer-specific pictures) used. This assumption aligns with previous studies that emphasized the importance of the strength of the stimulus in making fast and accurate decisions (Palmer et al., 2005). Hence, familiar stimuli are easier to process and effectiveness is even further increased for meaningful stimuli (Lupyan and Spivey, 2008). With regard to the sample of experienced soccer players, the soccer-specific images may have been of different meaning and thus, more relatable than the neutral objects in the computerized version. With regard to the different images in the tasks, the similarity of the images could also play a role here. The more similar the stimuli used, the more likely they are to be confused by the participants, thus, producing false alarms. However, more false alarms were produced in the computer task than in the soccer-specific task, although the images presented should be more clearly distinguishable than the images of soccer actions (for overview of all images used, see Supplementary material section C). Thus, it seems more likely that the shorter presentation of the stimuli and reaction time in the computer task provokes false alarms.

With regard to the considered influence of control variables, ambiguous results were shown. Motivation was significantly higher prior to the computer task than prior to the soccer-specific task whereas perceived exhaustion was higher prior to the soccer-specific task. Both results can be explained by the previously performed computer task itself. In other words, motivation decreased after the computer task and perceived exhaustion (mainly cognitive exhaustion also referred to as cognitive or mental fatigue; Sievertsen et al., 2016; Smith et al., 2018) increases which has been shown in previous research focusing on such laboratory tasks (O’Keeffe et al., 2020). There was moreover a negative correlation between exhaustion and response times in the soccer-specific task. Since the perceived exertion was assessed before the soccer-specific task and was significantly lower than before the task on the computer, it can be assumed that this is mainly cognitive exertion. Accordingly, the results are in line with studies on mental fatigue and its associated decline of soccer-specific performance factors including decision-making skills (Smith et al., 2016). Interestingly however, accuracy was higher in the soccer-specific task and thus, we would argue that this did not affect cognitive performance in the soccer-specific task.

Further, we found a positive correlation between motivation and response time in the soccer-specific task, indicating that higher motivation is related to slower response times in the soccer-specific task. However, as we did not measure motivation and perceived exhaustion after the soccer-specific task, and results actually indicated that players had more fun performing the soccer-specific task (see also Musculus et al., 2022), we would argue that the differences in prior motivation and perceived exhaustion to the tasks are negligible. Though, with regard to motivation and experienced fun, which correlated negatively to response time in the soccer-specific task, our results seem to contrast with findings showing that enjoyment leads to faster motor actions (see, e.g., Rathschlag and Memmert, 2013). However, results are hardly comparable as athletes had to react as fast as possible to presented stimuli in our study, thus they experience a cognitive load, whereas in Rathschlag and Memmert (2013), they had to throw a ball as fast as possible without responding to a stimulus.

Since this study primarily aimed at testing the validity of the developed soccer-specific *n*-back task, further investigations with regard to interindividual differences that could be reflected by performance of the task are relevant (see, e.g., Kalén et al., 2021). In this context, future research should investigate soccer players with different expertise levels and age groups as this might help clarify which cognitive functions are either developing (De Luca et al., 2003) or determined by expertise (Verburgh et al., 2014).

### Limitations

This study has some methodological limitations. The first is related to the study design: For each participant, the soccer-specific task was conducted after the computerized task. This could have potentially resulted in learning effects, which, on the one hand, were intended to reduce physical exhaustion and prevent injuries in the SoccerBot but, on the other hand, might have biased the results in the SoccerBot. However, potential learning effects within the two blocks of the respective tasks have not been identified. A further methodological limitation of the SoccerBot is the starting point for passing and response time assessment. The starting point from which the passes are played is located in the center of the artificial grass area, 5 m away from the screens. We have placed a marker and instructed the players to play from this spot, nevertheless, slight variations in movement execution are possible. Response times were inferred from a camera that assesses 120 frames/s (see also Musculus et al., 2022). Based on our results, however, the response time measures seem to be sufficient to detect variance in the response times similar to the computerized task. With regard to ecological validity of the developed task, we are aware that the representativeness of the applied stimuli is somewhat limited. The transfer of the required perception-action coupling for on-field performance is restricted as the players in the pictures often perform actions with a ball. In order to increase the representativeness of actual game situations, it would be conceivable to depict players in free spaces demanding the ball. The challenge here, however, is that differences between the images would only be marginal and could thus, easily lead to other methodological limitations such as an increase in false alarms. If the players in the pictures only differed in size and shape, it would be very difficult for the participants to distinguish and recall these differences in the short time span.

### Conclusion

In the present project, we aimed to develop a soccer-specific working memory task (*n*-back) in the SoccerBot100 and thereby expanding the repertoire of measuring soccer-specific inhibition and cognitive flexibility. Given significant correlations for response time and accuracy between the general, computerized and the adapted soccer-specific 3-back tasks, indicating convergent validity, we would argue that the task is applicable to measure soccer-specific working memory. Accordingly, together with the previously validated task, it is now possible to assess all three core EFs in a soccer-specific manner. Thereby, further investigations on the tasks’ external validation, for example in the context of other soccer-specific motor skills or the overall game performance are possible (see also Scharfen and Memmert, 2019; Heilmann et al., 2022). Finally, these further studies may allow conclusions to be drawn about the importance of EFs and enable to examine the expression of the individual EFs and their interaction.

## Data availability statement

The raw data supporting the conclusions of this article will be made available by the authors, without undue reservation.

## Ethics statement

The studies involving human participants were reviewed and approved by Ethics Advisory Board, Leipzig University. The patients/participants provided their written informed consent to participate in this study.

## Author contributions

SK, FL, LM, MR, and PW: idea. SK and FL: conceptualization. SK: planning of the study, literature research, data analysis, and first draft. SK and RS: data collection and data preparation. FL: revision and supervision. All authors contributed to the article and approved the submitted version.

## Funding

SK receives a promotional grant (LAU-R-G-31-1-1020) as part of the Saxony state graduate scholarship program (Landesgraduiertenstipendium) under the supervision of FL and A-ME. Publishing was funded by the Open Access Publishing Fund of Leipzig supported by the German Research Foundation within the program Open Access Publication Funding.

## Conflict of interest

The authors declare that the research was conducted in the absence of any commercial or financial relationships that could be construed as a potential conflict of interest.

## Publisher’s note

All claims expressed in this article are solely those of the authors and do not necessarily represent those of their affiliated organizations, or those of the publisher, the editors and the reviewers. Any product that may be evaluated in this article, or claim that may be made by its manufacturer, is not guaranteed or endorsed by the publisher.

## Footnotes

^1^We initially addressed inhibition and cognitive flexibility, which formed the basis for further research (for details on the procedure and the development process see: Musculus et al., 2022).
