# Supplementary material for: An assist for cognitive diagnostics in soccer (Part II): Development and validation of a task to measure working memory in a soccer-specific setting
Source: Front Psychol. 2023 Jan 23;13:1026017. doi: 10.3389/fpsyg.2022.1026017 (PMC9936861; doi:10.3389/fpsyg.2022.1026017)

Supplementary Material

# Supplementary Figures and Tables

1. Comparisons to Previous Working Memory Tasks

Table A.1

| **Authors** | **Participants** | **Objectives** | ***n-*back task Design** |
| --- | --- | --- | --- |
| Jaeggi et al., 2007 | Fifteen young adults  (9 women and 6 men, 21-29 years) | Examine individual differences in cognitive performance at the limits of working memory capacity | Stimuli: visuospatial stimuli consisted of blue squares, appearing in eight different loci on the computer screen  Levels of *N*: 0- back; 1-,2- and 3 back  Trials: 70 trials in the 0-back task, 40 trials in each 1-,2-, and 3-back task |
| Pelegrina et al., 2015 | children and adolescents (*N* = 3722, 1886 boys and 1836 girls) aged 7-13 years | Analyze age trend in n-back performance through childhood and early adolescence | Stimuli: 20 consonants  Levels of *N*: 1-, 2-, 3- back  Trials: 40 trials per level |
| Schleepen & Jonkmann, 2010 | Three groups of children (*N* = 57), one group of adults (*N* = 21) | Examine the development of WM-capacity and inference control interactions from childhood to adulthood | Stimuli: sequences of letters  Levels of *N*: 0-, 1-, 2- back  Trials: 360 in total, six blocks of 60 stimuli (two blocks per condition) |
| Shigeta et al., 2021 | Older adolescents from secondary schools  (*N* = 541, 43% female) | Investigate differential relationship of cardiorespiratory fitness, muscular fitness and cognitive control | Stimuli: series of shapes  Levels of *N*: 1- and 2- back  Trials: 2 blocks of 72 trials each for the 1-back and 2-back conditions |

*Detailed Comparisons to Previous Studies on the n-back Task*

1. **Detailed Description of the Soccer-specific Task in the SoccerBot100**

Figure B.1

*SoccerBot100*


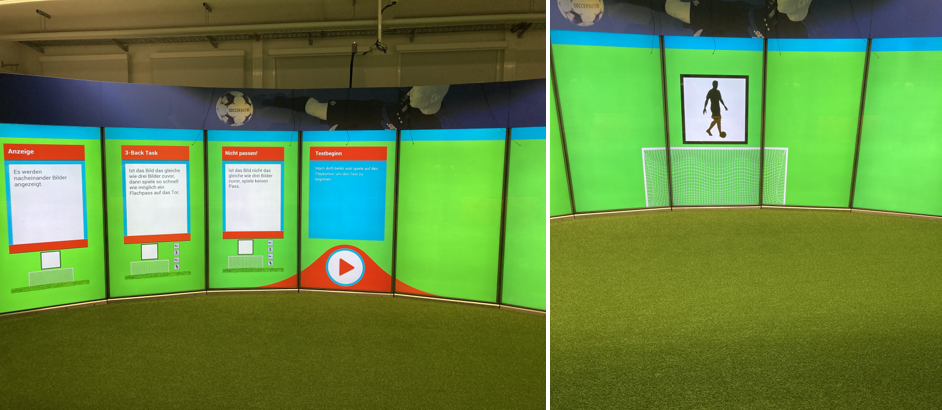


**Soccer-Specific task in the SoccerBot100**

The SoccerBot100 is a smaller version of the SoccerBot360 with a smaller field but with walls for projections and thus, providing the same technical functionality. The training content is shown on 7 full HD screens with a viewing angle of 100°. An integrated high-speed camera enables the recording of parameters like reaction time and processing time as well as passing speed or with which foot a player solves the given assignments through pass or shot. The playing ground is artificial grass. The starting point where the participants pass and control the ball is 5 m away from the screen. Therefore, all relevant features for the implementation of the cognitive diagnostics were identical to the Soccerbot360.

Instead of pictures of neutral objects, silhouettes with soccer specific actions were presented with a size of 1x1 meters. Therefore, one of the 8 monitors of the SoccerBot was used for stimuli presentation. Directly under the stimuli a goal was permanently presented. The representation of the goal stretched over three plates, with a width of 2.20 meters and a height of 0.95 meters.

1. Representation of the stimuli used

Figure C.1

*Images applied as stimuli in the computerized task*


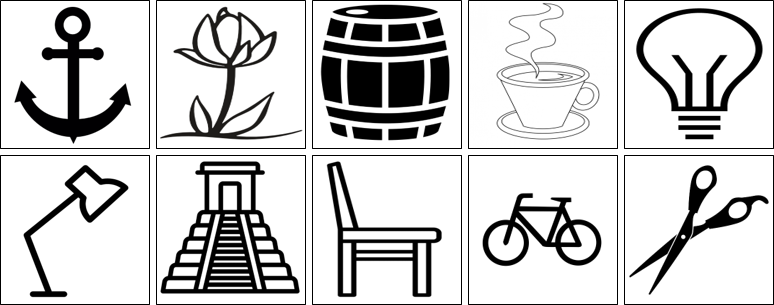


Figure C.2

*Images applied as stimuli in the soccer-specific task*


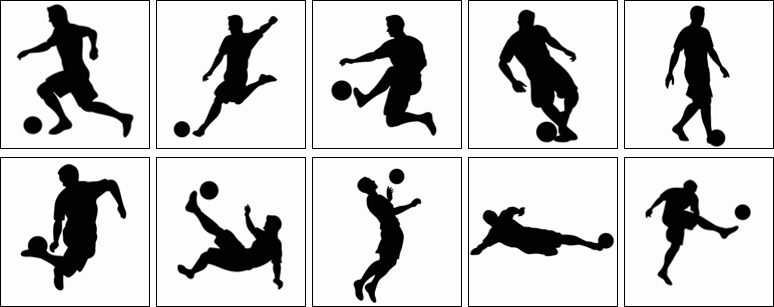

Supplement: Supplementary file 1 [file Table_1.docx]
